# The Treatment of Bubo

**Published:** 1883-05

**Authors:** G. Frank Lydston

**Affiliations:** Late Resident Surgeon Charity and State Emigration Hospitals, New York; Lecturer on Genito-Urinary and Venereal Diseases, College of Physicians and Surgeons, Chicago; 125 State St., Chicago


### ARTICLE V.

THE TREATMENT OF BUBO. By G. FRANK LYDSTON, M.D., Late Resident Surgeon Charity and State Emigration Hospitals, New York; Lecturer on Genito-Urinary and Venereal Diseases, College of Physicians and Surgeons, Chicago. (Read before the Chicago Pathological Society, March 12, 1883.)

The management of bubo, an affection which we are so frequently called upon to treat, even in general practice, is discussed in a very cursory manner by the majority of writers upon venereal diseases, and, I think, unjustly so, as the subject is a quite important one, and merits more attention than some of the rarer affections which are often allotted considerable space.

Notwithstanding the fact that bubo is a very common disease, it is only in large hospitals, in which there are wards devoted exclusively to diseases of a venereal nature, that we are able to make any accurate practical observations upon its management, for it is only under such circumstances that we have the patients completely under our control, and at the same time a sufficient number of cases to permit of the thorough application and comparison of the various modes of treatment. The results of treatment of isolated cases in private and dispensary practice are, to a great extent, modified by the habits and general conduct of the patient, which are quite frequently diametrically opposed to the directions of the surgeon, and may lead to the condemnation of really useful therapeutic measures. The patient, unless absolutely disabled, will not keep quiet; and not only this, but he will oftentimes continue to carouse, and, indeed, to keep up genital irritation, in spite of strict orders to the contrary, probably thinking, as too many are apt to think, that the various remedies prescribed ought to do their work without the slightest coöperation on his part. There are many points which must be mentioned in connection with the treatment of bubo, which are familiar, but which will bear repetition in the systematic discussion of its management. This is especially true of the elements of rest, counter-irritation and pressure. Regarding the subject from a systematic standpoint, the treatment of bubo resolves itself into several practical considerations: 1st, we have the question of prophylaxis; 2nd, the prevention of suppuration; 3rd, the management of suppurating bubo; 4th, the management of sinuses and exposed lymphatic glands; 5th, the management of gangrenous and phagedenic bubo; 6th, the management of chronic or indolent bubo.

With the exception of the prevention of suppuration and the consideration of the treatment of gangrenous and phagedenic bubo, these headings apply equally well to both the simple and virulent forms of the disease. The formation of pus cannot be prevented when a bubo is of a virulent nature, and a simple bubo is not very liable to either gangrene or phagedena, although either may occur under certain conditions.

The prophylaxis of bubo comprises but few points, but they are all important. The liability to the occurrence of bubo is of course greatly enhanced by any virulent property that may exist in the chancroid, if such be the source of irritation, and is of necessity, therefore, much greater in chancroid than in gonorrhœa, balanitis, or any of the simpler forms of local irritation, which so frequently give rise to adenitis.

Prophylaxis is consequently much less likely to prove effective in chancroid, and especially in its virulent form. Whatever the source of irritation, the chief prophylactic measure is, of course, perfect quiet, but in most of our cases of gonorrhœa or balanitic affections, and indeed, in chancroid, unless large and destructive, this is not practicable. We should, however, approximate it as nearly as possible. The patient may, at least, be impressed with the importance of avoiding all strains and violent efforts as far as possible. When a patient is compelled to go about during the progress of his venereal disorder, and especially if his occupation entails a certain amount of muscular effort, or prolonged standing at the desk or counter, an excellent plan is to apply a double spica bandage, with a compress in each groin, to prevent the injurious effects of strains by supporting the part. This should always be done on the first indication of inguinal irritation, unless the patient can rest for a time. Another essential feature of the prophylaxis is the maintenance of strict cleanliness of the genital lesion, thus avoiding the presence of irritating discharges. All irritating and cumbersome applications and dressing of the genital sores should be avoided as far as possible, and in case gonorrhœa exists, irritating injections should not be used. All greasy or fatty substances should be avoided, as a rule, in the treatment of genital lesions, as they readily decompose and become rancid, thus increasing the irritation already existing, and preventing the maintenance of cleanliness.

Ordinary ointments form such vile and filthy applications, that these remarks might seem superfluous, had I not seen several cases in which they had been used with deleterious effects, by different practitioners. When chancroid exists, the thorough destruction of the specific and virulent properties of the sore should be accomplished, thus obviating, as is generally accepted, the occurrence of anything but simple bubo, unless absorption of infectious material has already occurred, and greatly diminishing the liability to even that form.

This must necessarily be accomplished by the use of sufficiently powerful caustics. The substance used is of great importance. In spite of the advice of nearly all of our prominent authorities, I find that many practitioners use the nitrate of silver for this purpose. Now, in my opinion, this improper use of the nitrate of silver is a very fertile source of bubo, as it is but adding fuel to the fire. As a destructive cauterant under such circumstances it is worse than useless, as it sets up irritation, or even inflammation, without, as a rule, acting powerfully enough to destroy the specific characters of the sore. During the healing of the sore after its specific characters have been destroyed, the granulations may become sluggish ; as may happen in any simple ulcer, in which event the judicious application of nitrate of silver may be of great service. The same remarks will also apply to the sulphate of copper, which is sometimes used. Of the more powerful caustics, the actual cautery is, of course, the best, but it is not usually practicable to use it. The most generally used caustic is the fuming nitric acid. My own preference, however, is for the pure bromine. Either should be preceded by the pure carbolic acid, for the purpose of obtaining its anæsthetic effect.

I shall devote more attention to this subject in a subsequent paper, which I hope to be able to present upon the treatment of chancroid.

An important prophylactic indication is to maintain the free action of the bowels, as otherwise the straining during stool will produce inguinal irritation. I find that patients with bubo very generally complain of pain in the groins during a difficult stool. There is nothing more of importance to be said with reference to prophylaxis, and this brings us to the consideration of the prevention of suppuration, or the attempt to abort a threatening bubo. This is perhaps the most important element of our present subject in some respects. In the event that, in spite of prophylaxis, a bubo sets in, we should always, in my estimation, make the most strenuous endeavors to prevent the formation of pus, as the healing of a bubo after incision or rupture is usually a matter of considerable time, and often is exceedingly slow, even in the best hands, aside from its liability to serious inflammatory or hæmorrhagic complications. In addition to these disagreeable features, there is the usual result of a very unsightly discolored cicatrix, a by no means unimportant consideration, especially in women. The anti-suppurative treatment of bubo comprises several measures. We have first that which is perhaps most frequently used—counter-irritation, either with or without pressure. This method and its principles are quite universally understood, and will require comparatively little comment. The counter-irritant most frequently used is the ordinary tr. of iodine; or, better, the compound tr. with an extra amount of iodine added. If combined with pressure, a shot bag of say five pounds weight, is an excellent method for its application.

A better plan for the use of pressure, however, is to apply a spica bandage, over compressed sponge, which is laid upon the bubo, the sponges being subsequently kept wet with cold water. As the sponge swells, we have a very firm and equable pressure exerted upon the tumor, in addition to the antiphlogistic effects of cold.

The benefits derived from this form of treatment are explained by the local anæmia thus produced, and the prevention of further exudation. A very good method of applying pressure is by the free application of collodion. The bubo is first thoroughly painted with the tr. of belladonna, and as soon as this is dry, the collodion should be applied. This should be repeated daily. I have seen this plan act very well. A combination of the use of belladonna plaster and pressure is also recommended. Blisters are sometimes used in this stage of bubo, but are not effective in producing anything but discomfort. The application of leeches for the purpose of averting suppuration by causing local depletion, is, I think, to be deprecated, as the leech bites form lesions capable of becoming infected by auto-inoculation in case the bubo should prove to be a virulent one, and the fewer such lesions the better. The injection of carbolic acid into the substance of the inflamed gland is strongly recommended by Dr. M. K. Taylor, U. S. A., as a method of aborting bubo, but it does not seem to be as successful in other hands as it would appear to have been in those of its originator. My own experience with it is quite limited, but the few cases in which I have given it a trial have not afforded me any encouragement. It could have no effect in virulent bubo in any event, and its antisuppurative power in even simple inflammation is, to say the least, doubtful. The mode of procedure is to make a number of injections into the substance of the inflamed gland of a 1-15 solution of ac. carbol. Busch, of Bonn, recommends Kern's cataplasm, which is composed of black soap and mustard, one-fourth or fifth. I have had no experience with it, but the rationale of its action is such that I am inclined to deem it worthy of trial. Mercurial inunctions are sometimes resorted to but in acute bubo should never be used, as, in common with all other applications requiring friction, they are injurious. In other phases of bubo we will hereafter consider their use. The iodide of lead ointment, in combination with the extract of belladonna, is oftentimes successful in aborting a bubo, if simple, and in diminishing the surrounding inflammation, if virulent, although it does not in the latter case prevent suppuration. I prefer this plan to the application of the acetate of lead as recommended by Ziessl and Patzelt.* Ziessl's method is to soak a number of compresses in a solution of the acetate of lead, and bind them upon the bubo, keeping them thoroughly wet. The ordinary lead and opium wash, with an increased proportion of both ingredients, is probably much better than a simple solution of the acetate. This was a favorite application with some of my hospital associates. Punctate cauterization is recommended by Vidal, but I do not think much of its usefulness in the acute form of bubo. A modification of it I am decidedly in favor of in the chronic forms, and will mention it in that connection. We now come to what, in my opinion, is the most powerful anti-suppurative measure at our command, the much-used and little-understood poultice. It is not long since such a claim for this remedy would have been greatly ridiculed, and it is even now held to be absurd by many practitioners. There is little said by those who advocate the use of poultices and hot fomentations in inflammation, in explanation of their action, save that they have an emollient or sedative effect, and favor free circulation, and it will perhaps not be out of place to attempt to explain, in some measure, the diametrically opposed effects of these applications. In the first place, it will be necessary to give a short description of those pathological changes in the tissues, which constitute an abscess, whether from bubo or other causes. As a result of inflammation, we have a localized accumulation of leucocytes in the inflamed tissues, these leucocytes, according to our best pathologists, having several sources for their production, viz: 1st, migration of white blood cells from the blood-vessels, notably the veins, into the tissues; 2nd, the leucocytes subsequently multiply by division, thus further increasing the amount of the purulent formation; 3rd, localized proliferation of the cellular elements of the connective tissue. As a result of the accumulation of cells, we have tension of the tissues, varying in degree with the amount of the corpuscular elements; there is also a certain amount of fluid transudation, constituting the liquor puris.

* *Archiv. für dermatologie und syphilographie Pvag.,* 5 *Jahrg.,* 1873-4 *Heft.*

There is in all inflammations profound circulatory disturbance, and at the period of purulent formation, this chiefly consists in obstruction and stasis, which is still further increased by the circumscribed collection of cells, to a degree proportionate to the tension present. As a result of the pressure, there is lymphatic obstruction, and consequent abeyance of the function of the absorbents. The vitality of the tissue elements is impaired to an extent greatly modified by the amount of pressure and circulatory disturbance, this impairment of nutrition being greatest in those tissues immediately contiguous to the accumulation of pus, and shading off into the surrounding tissue area. As a result mainly of the pressure of the purulent formation, a layer of partially organized lymph forms upon the tissues surrounding the abscess cavity, and this in chronic abscess forms a pseudo membrane, which has erroneously been termed the "pyogenic membrane."

In acute abscess, however, it simply shades off into the surrounding tissues, which are, in a measure, matted together by the inflammatory exudate. The thickness and degree of organization of the layer of lymph, and the extent to which the vitality of the surrounding tissues are impaired, which, as I have said, depends mainly upon the amount of inflammatory exudate, and the consequent disturbance of the circulation, determines the facility with which resolution of a circumscribed inflammation on the one hand, and the formation of pus upon the other, takes place. It has been very generally claimed that pus, when once formed in a circumscribed collection, cannot be absorbed, but this is erroneous.

It is well known that the first factor in the production of the phenomena of inflammation, is the existence of irritation, and that this results in the various changes which I have described. This irritation is augmented still further by the accumulation of inflammatory products. Now, when we apply a hot poultice to an inflamed part, we first relieve the irritation and pain, which has an immediate effect in preventing or lessening further exudation, and probably produce a certain amount of vascular contraction, with consequent anæmia, through the medium of the vaso-motor nerves. By lessening the amount of exudate, we lessen the amount of circulatory obstruction, and thus relive stasis and diminish the impairment of nutrition resulting both from pressure and stasis. Relief of the circulation by lessening pressure is also attended by relief of obstruction, and the restoration of function in the absorbents, which changes are necessary for resolution.

If the inflammation has been very severe, and the exudation great, the nutrition of a certain number of the tissue elements has been so impaired that they cannot be resolved, and resorption, which readily occurs when the vitality of the tissues is only moderately impaired, cannot then occur. In an instance of this kind, the application of moist heat will fail to prevent suppuration, but will limit the amount of exudate, and prevent further tissue change. There is a certain area of hard indurated tissue about an abscess, as is well known, which area represents that, the changes in which I have described. According to the view I have presented, the cells in the outer portions of this area may have sufficient vitality to become resolved, while those in immediate relation with the abscess cavity have gone too far for such a change to occur. It is an observation that any one may verify, that the less the amount of induration surrounding an abscess, and the sooner it resolves or breaks down into pus, the sooner will the abscess heal after it is incised. There is much of truth in the popular idea that an abscess must be "ripe" before it is opened. Healthy granulations cannot spring up from tissues whose vitality is impaired by the pressure of a large amount of inflammatory exudate. The distinctness of fluctuation which is so evidently increased by poulticing, depends upon the amount of surrounding exudate, and at the same time that the tissue over an abscess is becoming thinned by the pressure of the pus, the inflammatory exudate in its meshes is being removed by the action of the poultices. We may formulate the action of moist heat, then, by saying, 1st. That it will prevent the formation of pus if the vitality of the tissue elements has not become too greatly impaired. 2d. That it will hasten maturation, and limit the purulent formation, if the impairment of nutrition has gone too far to permit of resolution. 3d. That it will diminish the indurated area about an abscess after incision or spontaneous rupture, and favor healthy granulation. It may be accepted, therefore, that poultices, or the application of moist heat in some form, are beneficial at any stage of inflammation and abscess. Such applications, it is true, may be continued too long, with the effect of relaxing the tissues to such an extent that they become boggy and infiltrated, but practical experience alone will teach as how to avoid such a contingency. When we speak of the beneficial effects of poultices in the treatment of inflammation, whether of a lymphatic gland or any other tissue, we assume that such measures are properly applied, and not in the slipshod way which seems to be the rule. They are not, as a rule, properly made to begin with, and if hot at the outset, are quite likely to be allowed to become cool before they are applied to the part. Once applied, they are usually allowed to remain upon the part until they are cold, thus neutralizing any beneficial effects which may have been attained by the heat; if, indeed, the inflammation has not been augmented by the clumsy manipulation, as well as the rapid changes of temperature to which the part has been subjected.

As many practitioners, even, are lame in the apparently trivial question of poultice-making, or, if well versed in this, are in the habit of leaving the matter to the nurse, a few words upon this subject may not be superfluous. An emollient poultice, to be of service, should be sufficiently large, whatever its composition, to extend beyond the borders of the inflamed area; in short, should be a generous one. The material should be gradually stirred in boiling water, until of the proper consistency, and should usually be made fresh for each poultice, and not set aside to become caked and lumpy, as a poultice of this kind is very uncomfortable and irritating. The batter should be spread upon thick muslin, in a layer of one-half to three-fourths inch in thickness, a space of an inch or so being left at the edges. If the inflammation be very painful, a few drachms of laudanum may be sprinkled upon the poultice. The whole is now covered with a layer of cheese-cloth, and the free edges turned over and secured in such a way that the poultice material will not ooze out and soil the person and clothing of the patient. It should now be quickly applied, and covered with oiled muslin. Thus made and applied, a poultice forms a very neat application, but as ordinarily constructed, the patient and his clothing get besmeared in a very disagreeable fashion. A very convenient plan, especially in bubo, is to have a number of muslin bags of the required size prepared, to be filled and applied as required. A poultice should be renewed sufficiently often to keep it hot, or no benefit can be expected. A convenient method of obviating the necessity of making a fresh poultice for each application, is to prepare a number, and keep them hot by means of the ordinary utensil for steaming bread. After a bubo has been opened, antiseptic poultices may be necessary, and in such an event, equal parts of charcoal and linseed meal, mixed with hot yeast instead of water, form the best material.

The next anti-suppurative measure worthy of attention is the internal administration of the calx sulphurata, or, as it has been erroneously termed, the "sulphide of calcium." The pure sulphide of calcium is not found in the drug market, nor has it ever been used therapeutically. Calx sulphurata, or the sulphurated lime, as described by the new pharmacopœia, is a mixture of the sulphate and sulphide of calcium in varying proportions, and containing not less than 36 per cent. of the latter in pure form. This drug has been highly lauded as an antisuppurative, and has been especially recommended in the treatment of bubo. Otis, in particular, has praised its action in this affection.

Although the drug has been greatly overrated by some, and unjustly condemned by others, I am of the opinion, derived from a considerable experience in its use, that it is a remedy of great value, and that it will produce several marked effects, varying with the character of the inflammation. Its action in the prevention of suppuration is similar to that of poultices, and like the latter, it will, unless the inflammatory changes have gone too far to permit of resolution, favor suppuration and hasten maturation. The rationale of its action is probably the same as that of moist heat, with the exception of the absence of the local sedation produced by the latter. It also, I think, acts by producing fatty degeneration of the inflammatory exudate, and thus relieving the circulation, or in a manner analogous to mercury. Unlike poultices, it will cause pus which is already fully formed in a circumscribed cavity to become absorbed, probably through this same fatty degeneration. The possibility of this is by many denied, but I have seen many instances of such action. One of these I

will cite, as especially striking. A pale, lymphatic female entered my ward in the hospital, suffering from a slight vaginitis, probably gonorrhœal, and presenting in the left groin a suppurating bubo, of a particularly chronic character, there being distinct fluctuaation throughout, and no redness or induration about it. The tumor was as large as a good-sized hen's egg, and the skin covering it was so thin, that I expected it to burst spontaneously during the night after her admission. On the next day, I was about to incise it, but the woman was so anxious to avoid a scar, that I put her upon one-half gr. doses of the calx sulphurata every three hours and deferred the operation.

Much to my surprise, the pus entirely absorbed in less than a week. The dose of the drug should vary with the stage of the inflammation. Where it is desired to prevent suppuration, the dose should be from one-twelfth to one-tenth gr. every hour. When, however, the inflammation has so far advanced that suppuration is inevitable, the dose should be increased to about one-fourth to one-half gr. every three hours. In chronic and indolent bubo, and in cases in which unhealthily secreting surfaces or sinuses are left after incision or rupture of the abscess, the last-mentioned doses will often speedily bring about a healthy action, the surrounding induration rapidly disappearing, and the character of the pus changing from sanious or ichorous to a free laudable secretion, after which granulation is quite rapid. The great variance of opinion as to the effect of the calx sulphurata in inflammation is only explicable by the ignorance that prevails in respect to its proper use. Like moist heat, this drug, in proper doses, will produce beneficial effects at any stage, or in any variety of bubo. As much as has been said of the antisuppurative treatment of bubo, we are compelled to acknowledge that it is only effective in simple bubo, and that the virulent form must inevitably suppurate, but as our anti-suppurative measures may, under certain circumstances, also promote maturation, and will always limit the surrounding inflammation, they are always indicated. Again, it is not always possible to affirm that a bubo is virulent prior to suppuration. If, however, the primary lesion be an auto-inoculable chancroid, and the resulting bubo runs a very acute course, we are warranted in assuming that it is virulent. After the bubo is opened, the diagnosis is, of course, quite easy.

The importance of constitutional measures in the management of bubo can scarcely be overrated, and may properly be alluded to in connection with the anti-suppurative treatment of the disease. As soon as bubo threatens, the derivative effect of free cathartics should be obtained, and throughout the course of the affection mild cathartics should be given, for reasons stated in connection with the subject of prophylaxis. If the patient be at all debilitated, tonics should be freely given, and if struma be evident, cod-liver oil and the syr. ferri iod. should be administered. As a rule, too much dependence is placed upon local measures, and too little attention given to the constitutional condition. How often do we see a chronic, indurated, open bubo heal in a short time under proper measures of constitutional treatment. There is one point with reference to the prevention of suppuration in bubo, which it may be well to mention. We will find a great many patients who object to any measures which are calculated to "scatter" the bubo, on the ground that such a plan "drives the poison into the blood," and we will also find that if we succeed in aborting the bubo, all subsequent eruptions of the skin, and perhaps other troubles, will be laid at our doors. Their objections are mainly to such measures as they can understand to be anti-suppurative, such as the application of counter-irritation and pressure. There might be some foundation for this popular notion, if it were possible to discuss a virulent bubo. When we find a patient of this kind, however, we should do our utmost to promote suppuration, hoping that the resulting scar will be sufficiently large and unsightly to give satisfaction.

The treatment of suppurating bubo is the next topic for consideration, and has been indicated to some extent in the discussion of the action of moist heat and calx sulphurata. When we find that suppuration is inevitable, which is always the case in virulent bubo, we should at once endeavor to promote the formation of pus by every means in our power. If poultices have not already been employed, we should at once apply them, in conjunction with hot fomentations. If calx sulphurata has already been used, we should now increase the dose to its maximum, which should ordinarily be about one-fourth to one-half gr. every three hours. Larger doses may, however, be given. As soon as fluctuation is distinct, and the surrounding induration has in some measure disappeared ; in short, as soon as the abscess is "ripe", it should be opened. An early opening is essential in virulent bubo, as we are very apt to have burrowing in this form. We have already seen that when a simple bubo is opened too early, the process of repair is apt to be very slow. There are exceptional instances in which other measures than incision are best in the treatment of suppurating bubo, but these are cases of the subacute or chronic form, which we we will consider later on.

The manner of opening a suppurating bubo is of great importance. As a rule, the operation is only half done, a simple small incision being made, which is barely sufficient to give exit to the contained pus, and entirely inadequate to permit of the proper cleansing of the abscess cavity. If the bubo be virulent, troublesome and extensive burrowing is apt to occur. The only way to prevent this, is to lay open all sinuses and pockets thoroughly. As soon as the pus is evacuated, the abscess cavity should be washed out with a five per cent. solution of carbolic acid, or some other disinfectant solution, and if at all virulent in appearance, the pure carbolic acid should be thoroughly applied by means of a swab, and should be carried to the bottom of all sinuses and depressions. The edges should now be thoroughly cut away, if at all undermined, and the cavity converted into the shape of a saucer, as nearly as possible. The cut surfaces will require the pure carbolic acid to prevent infection. The peroxide of hydrogen, a substance which has recently come into great favor as an antiseptic, is useful as an application to both virulent bubo and chancroid, apparently destroying the specific properties of these lesions, and setting up healthy action in the most remarkable manner. It may, therefore, oftentimes answer the purpose of cauterization with more powerful irritants, or detergent and antiseptic substances. Not more than two or three applications are necessary, the sore taking on healthy action quite readily under this remedy.

When a bubo is of a simple inflammatory nature, which may usually be determined by the character of the pus, and the history and course of the inflammation, cauterization is unnecessary, and after having been thoroughly cleansed, the cavity should be packed with picked lint saturated in a 1-20 sol. of carbolic acid. If there be much surrounding induration, or if the flow of pus after the incision should be scanty, poultices should be applied. Virulent buboes should be packed with equal parts of boracic acid and iodoform, and a hot poultice applied over all. I much prefer the measures I have suggested, to the routine process of packing the abscess cavity with oakum and bals. Peru. In a few days, however, after the bubo has taken on healthy action, or if simple, and it requires gentle stimulation, the pure bals. Peru. may be applied on picked lint, not oakum. My preference is for the following formula, rather than the clear balsam:

℞. Iodoformi..........................ʒii.

Ac. boracici........................ʒii.

Bals. Peruviani.................. ℥i.

Vaselinæ ad...................... ℥ii.

M. Sig. Apply upon picked lint.

When an application of a more stimulating nature is required, the following will be found useful:

℞. Argenti nitratis.................. ℈i.

Pulv. stramonii fol............... ʒii.

Ext. belladonnæ..................gr. v.

Cerati simplicis ad............. ℥i.

M. S. Apply.

The solid stick of silver may be required from time to time, as in ordinary processes of granulation. When a bubo is sluggish and secreting unhealthily, I have found a powder of equal parts of oxide of zinc and red cinchona bark to act remarkably well. The advantages of removing the edges of the bubo after incision, and converting it into a saucer-shaped cavity, are several. In the first place, it summarily disposes of the edges, which so frequently tend to invert, and almost invariably become indurated and thickened, thus preventing healing. 2d. It facilitates cleanliness, and enables us to make our applications to all parts of the cavity. 3rd. It prevents burrowing, and enables us to remove projecting glands with great facility, and also imparts to the bubo many of the characters of simple ulcers in other situations. 4th. It favors rapid healing from the edges, as well as from the bottom, and leaves a much less puckered and discolored scar than when the edges are left. Of the various methods of evacuating a suppurating bubo without free incision, such as multiple puncture, aspiration, and Auspitz's method of breaking up the inflamed gland by means of a blunt probe introduced through a small incision, I will simply express the opinion that, as a rule, they must be followed by free incision, sooner or later, if the bubo be virulent, and with the probable result of finding that burrowing to a greater or less extent has occurred. It is, of course, desirable to avoid a scar if possible, and in simple bubo, this may sometimes be done, especially by aspiration, and the use of calx sulphurata, but, as a rule, the plan that I have mapped out will be found to yield the best results, and I have yet to see an evil result from it, in a large experience in the treatment of bubo. Opening buboes by caustics is very unsurgical, and not to be considered, for we may resort to local anæsthesia if the patient be nervous, and dreads the cutting. I have found the application of pure carbolic acid to answer this purpose very well, in lieu of the ether or rhigolene spray. In connection with the subject of suppurating bubo, I will take the opportunity of mentioning a method of treatment recommended by Kümmel.* This gentleman uses a dressing of a solution of the bichloride of mercury after the evacuation of a bubo. He extirpates the whole group of glands, as well as those affected. After the removal of the glands, the wound is dried with sponges, drainage tubes are introduced, and the wound sutured throughout its extent, save for a short space at the most dependent portion, which is left free for drainage. The wound is now covered with compresses of gauze saturated with the solution of the bichloride, or with small ash bags, the whole being covered by larger bags. Firm compression is next obtained by a roller bandage, and the dressing is then allowed to remain undisturbed for eight or ten days, at the end of which time it may be renewed, if necessary. The wound is kept quiet by short splints applied to the thigh and pelvis. When ulceration exists, all the infiltrated tissues are scooped away, and the cavity thus formed packed with sand wet with the bichloride solution, over which layers of gauze are applied.

---

* *Centralblatt für Chirurgie*, Dec 30, 1882.

If the discharge be free, the gauze is changed as required, and the sand again wet with the mercuric solution. Good results are claimed for this plan by Kümmel, and he states that if no ulceration has occurred, he obtains union by first intention, and where an ulcer has resulted, he claims that granulation is rapid, and suppuration slight. I have had no opportunity as yet to try this method, and am hardly prepared to express an opinion as to its merits, but as regards simplicity, I think that it hardly compares with the method which I have described. I should hardly be willing to suture the cut edges after the incision of a bubo, which may present no virulent characters when first opened, but which may subsequently assume the characters of chancroid, and perhaps burrow quite extensively. The open treatment is devoid of these dangers, especially if the edges be thoroughly removed. There are no foci for auto-infection in this plan, as are afforded by the suture tracks in the method of Kümmel.

The management of sinuses and exposed glands requires but little attention, or a few words, at least, will suffice for the consideration of the most valuable measures for their treatment. If a bubo be properly opened, and the undermined and degenerated tissue at its edges thoroughly removed, sinuses are not apt to form, but we will often meet with cases that have been improperly handled, in which sinuses of greater or less number and extent have resulted. When practicable, such sinuses should be thoroughly laid open, and the hard and indurated track cut away. They may sometimes be induced to heal by applications of the solid stick of the nitrate of silver, but they are quite liable to reopen, especially if the patient is cachectic, or moves about a great deal, as the tissue about them is of a very low grade of vitality. When too deep to be freely laid open, or when they are in dangerous proximity to important structures, they may often be induced to granulate from the bottom, by keeping them freely open with sponge tents, and stimulation with caustics. An excellent plan for deep sinuses is that often used for sinuses and fistulæ in other situations, viz: incision of the external opening, and the insertion of a wedge-shaped piece of wax, the base of which is gradually shaved off as the bottom of the cavity granulates. Injections of very hot water, frequently repeated, have also proved quite useful in my own practice. I usually combine them with the use of pencils or tents of iodoform, and it is this plan which has afforded me the most favorable results. The tent is to be dipped in vaseline, and then inserted into the sinus, care being taken that its bottom is reached. It is then cut off level with the surface, and powdered iodoform and a compress applied over all. I have also used a mixture of iodoform and glycerine, ʒii to the ounce, as an injection for sinuses and fistulæ in varions situations, and have had excellent results.

The employment of sponge grafting would seem to offer promising results, reasoning from the success reported by those who have used this method for the treatment of ulcers and sinuses of various kinds not dependent upon bubo. The sponge is to be washed in a solution of dilute muriatic acid, and then carbolized with a solution of carbolic acid, 1-20. It should be packed tightly in the sinus, and covered with a compress saturated in carbolic acid. The subsequent discharge, which is usually quite free, is kept from accumulating by the free use of a carbolized solution similar to that used for saturating the sponge. In a short time, it is claimed, granulations will spring up, and the sinus rapidly heal. The method of sponge grafting is also applicable to the treatment of open bubo without sinuses, which may become sluggish and indolent. Sinuses will often take on a healthy action, and heal, under full doses of calx suppurata.

The management of exposed and hyperplastic glands ought to be sufficiently simple. When free glands are found on opening a bubo, they should at once be removed, for, if left, they will, as is well known, act as foreign bodies, and prolong the healing process indefinitely. In many cases, the fingers will suffice for their removal, but if not, Volkmann's spoon or Piffard's curette may be used, or they may be ligated with silver wire, and allowed to separate spontaneously. A plan sometimes used, and which I have myself practiced, is to destroy the glands by repeatedly boring them, so to speak, with a sharp point of pure nitrate of silver, but I will take the present opportunity of deprecating this practice, which is very tedious and painful. In some cases, the glands are not found to be free when the abscess is first opened, and in such instances they should be allowed to remain a few days, until they become somewhat enlarged and distinctly outlined, at which time they are easily removed.

A very important point in the treatment of open bubo, is the question of constitutional syphilis. If a genital sore be of the mixed variety, the resulting bubo is quite likely to heal very slowly, if at all, unless a course of mercury is administered, and where the patient has had syphilis a certain length of time prior to the occurrence of the bubo, he will also require a full mercurial ceurse. If the attack of syphilis be somewhat remote, or if the patient be greatly debilitated, and suffering from the syphilitic cachexia, an antisyphilitic course of mixed treatment on the one hand, or of small tonic doses of mercury upon the other, will be required. The necessity for antisyphilitic remedies in those rare instances of suppuration following purely syphilitic adenopathies, is too obvious to require more than a mere allusion. In the cases of syphilitic cachexia that I have mentioned, the administration of small tonic doses of the bichloride of mercury will often operate in a remarkable manner, a bubo which has run a very prolonged course healing quite rapidly, and the general health of the patient improving in a marked degree. This tonic action of mercury is hardly appreciated, I think, by the majority of even those who employ mercury quite extensively, in spite of the conclusive experiments of Keyes, and his remarkable results with the hæmatometer.

The treatment of bubo, complicated by gangrene or phagedena, does not differ from that of chancroid attended by the same complications. Much may be done to prevent these disagreeable and serious complications of bubo, by proper attention to the constitutional condition of the patient. If cachectic or debilitated, he should be put upon tonics and a highly nourishing diet at the outset. There is nothing better as a tonic under such circumstances, than the potassio-tartrate of iron, a remedy highly extolled by Ricord in the treatment of phagedena. As is well known, however, we occasionally meet with cases in which phagedena occurs without any evident cause, and in which we are compelled to recognize an innate predisposition to the affection.

When phagedena or gangrene attack a bubo, the first indication is the thorough destruction of the diseased surfaces by cauterization. This should not be done in a feeble, halfway manner, or it will be ineffectual, or even injurious, and will require repetition. An anæsthetic should always be given, if the diseased surface is at all extensive, or the work is not apt to be thoroughly done. The caustic used is greatly a matter of choice, providing one be used which is sufficiently powerful to destroy the tissues for the required extent. The Paquelin thermo-cautery, pure bromine, or Ricord's paste, may be used, but I think that the bromine will prove the most satisfactory. After the operation, an antiseptic poultice should be applied, and morphia freely given to alleviate the pain, which is sometimes considerable. When the carbo-sulphuric paste is used, the patient should be kept well under the influence of opium during its application. Opium has been said to have in itself a somewhat specific action in checking phagedena, aside from its mere narcotic effect.

When it is not practicable to cauterize the surfaces, iodoform, carbolic acid, the peroxide of hydrogen, and the potassio-tartrate of iron in a solution of gr. xl. ad ℥s. have each their advocates as local applications. My own preference is for the peroxide of hydrogen, followed by close packing of the cavity with pure iodoform or boracic acid.

Some cases of phagedena, notably the serpiginous form, will progress with greater or less rapidity, in spite of the very best treatment, and others, after the phagedena has been checked and the sore is very nearly healed, will suddenly take on phagedenic action. Simple bubo may do the same thing, in rare instances. I recall one case occurring in the ward of my friend, Dr. Frank W. Merriam, then house-surgeon at the Charity Hospital, in which the bubo was apparently nearly closed, and was granulating finely, when gangrenous phagedena set in, and extended over nearly half the abdomen, before it could be checked. By free stimulation and a nourishing diet, with the local application of the carbo-sulphuric paste, the process was finally stopped, but not until the external oblique muscle had been destroyed for quite a space, and even the next layer of muscles attacked. This case was primarily one of virulent bubo, but had not been phagedenic, and at the time the phagedena set in was practically a simple bubo in process of granulation.

Vidal claims to have used the pyrogallic acid in the treatment of phagedena with considerable success.* It is claimed to destroy the auto-inoculability of the sore, and to bring about a healthy action in a remarkable manner. It may be used either as an ointment composed of one part of the acid to two of vaseline, to be applied by the surgeon, or as a powder composed of one part of the acid to four of starch, which the patient may apply himself. The pyrogallic acid gives a blackish color to the part, which is of no special signification. I have not yet had the opportunity of trying this treatment in phagedenic bubo, but have used it in several cases of simple and phagedenic chancroid, with good effect.

Chronic and indolent bubo, whether with or without the formation of pus, is usually found in persons of a strumous diathesis, or those who are debilitated or cachectic from some cause. Where suppuration occurs in such cases, the bubo may run an ordinary acute or subacute course, but after the evacuation of the pus, it takes on the characters of a chronic and indolent ulcer. Phagedenic bubo is apt to become chronic, and last indefinitely.

The chief measures of treatment in chronic and indolent bubo, occurring in scrofulous or cachectic subjects, consist in the administration of such remedies as the iodide of iron, cod-liver oil, arsenic, iodoform, or, in short, any of the remedies of known antistrumous power, as well as such ordinary tonics as quinia, or the mineral acids. A liberal diet, of which milk and cream should form the principal ingredients, and improved hygiene, are usually called for. It is in just such cases as those at present under consideration, that the sulphurated lime will yield the most brilliant results; no better, however, than in strumous affections of other varieties. The maximum dose should be given. As illustrated by a case which I have already cited, absorption of pus may be brought about by this drug, after it has fully formed, and in considerable amount. If the bubo goes on to suppuration in these chronic cases, aspiration may be used in conjunction with the sulphurated lime, and in this way a cure may sometimes be

* *Journal de Médecine et de Chirurgie.*

effected without the necessity of producing a scar by a free incision.

Chronic bubo may remain hard and indolent for a long time, before pus forms, and various local measures are sometimes useful in bringing about resolution without suppuration. The different methods of counter-irritation and pressure already described have numerous advocates. I have mentioned the method of punctate cauterization, in connection with acute bubo, as applicable to the treatment of the form at present under consideration. The modification of this method which appears to me most effectual, consists in drawing a series of intersecting lines over the surface of the tumor with the Paquelin cautery, in the manner often used in inflamed joints. Although not very painful, this method is usually objected to by the patient. Erichsen has found the discutient method of Malplaquet to be very serviceable in chronic and indolent bubo. This consists in the application of a blister of the size of a half-crown to the surface of the enlarged gland, and subsequently dressing the raw surface with lint soaked in a saturated solution of the bichloride of mercury. At the end of two or three hours, a white eschar will have formed, and the lint is then to be removed, and cold dressings applied. Simple blisters are useful also in chronic bubo. They should be repeated sufficiently often to keep the surface raw, and a dressing of the oleate of mercury or blue ointment kept constantly applied. An ointment of equal parts of the oleate of mercury and compound iodine ointment is often effectual in producing resolution without the previous application of a blister. Injections of arsenic have been found useful in strumous and other glandular enlargements in other situations, and would consequently be worthy of trial in chronic bubo.

There is one form of chronic bubo which merits especial attention. This is the variety which accompanies the form of chronic chancroid termed "lupus of the vulva," or in the male, chronic phagedena. This form of bubo is identical in its general characters with the lesion of the genitals, and presents an elevated, hyperplastic mass of tissue of greater or less extent, with an unhealthy pultaceous or worm-eaten appearance of its surface, which secretes an unhealthy, ichorous fluid. The disease extends very slowly, if at all, after having attained a certain size, the ulceration having meanwhile become continuous in many cases with the genital ulcer. There are apt to be several of the buboes, either distinct, or connected by ulceration. Such cases are very apt to be of a hæmorrhagic nature when they occur in pregnant females. Cases of this severe form of chronic bubo are probably never seen in private practice, but are found only in broken-down hospital cases. They will often defy the best measures of treatment, and finally wear the patient out.

When this form of bubo refuses to yield to the ordinary local treatment and the usual routine system of tonics and dietetics, the occasional application of the actual cautery will sometimes excite a healthy action, with active granulation and repair. As a dressing, iodoform is probably the best substance. An infusion of cinchona bark may also be of service, a piece of lint being saturated with it and laid upon the part, to be subsequently wet sufficiently often to keep it moist. Bumstead recommends the pure persulphate of iron in these cases. Judging from my own experience, the management of the affection is anything but satisfactory.

125 State St., Chicago, March 10, 1883.

---

MORTALITY OF THE GLOBE.

The *Gazetta Medica*, of Petersburg, makes a curious calculation on the mortality of the terrestrial globe. Europe contains 309,000,000 of inhabitants, Asia 804,000,000, Africa 199,000,-000, America 85,000,000 and Australia 4,500,000: total, 1,451,500,000. Taking, as a basis, the average mortality of France, comparatively a moderate proportion, on account of the good hygenic and climatological conditions of that country, there occur annually 35,692,835 deaths in all the terrertrial globe, or 97,790 per day. The number of births are 70 per minute, and 104,800 per day.

